# Gtpbp2 is a positive regulator of Wnt signaling and maintains low levels of the Wnt negative regulator Axin

**DOI:** 10.1186/s12964-016-0138-x

**Published:** 2016-08-02

**Authors:** William Q. Gillis, Arif Kirmizitas, Yasuno Iwasaki, Dong-Hyuk Ki, Jonathan M. Wyrick, Gerald H. Thomsen

**Affiliations:** 1 Department of Biochemistry and Cell Biology, Graduate Program in Molecular and Cellular Biology, Center for Developmental Genetics, Stony Brook University, Stony Brook, NY 11794-5215 USA; 2 Present Address: Department of Biological Sciences, State University of New York, College at Old Westbury, Old Westbury, NY 11568 USA; 3 Present Address: The Weatherall Institute of Molecular Medicine, University of Oxford, John Radcliffe Hospital, Headington, Oxford OX3 9DS UK; 4 Present Address: Department of Pediatric Oncology, Dana-Farber Cancer Institute, Division of Hematology/Oncology, Children’s Hospital Boston, Harvard Medical School, Boston, MA USA

**Keywords:** Axin, GTPase, Gtpbp2, Wnt signaling, *Xenopus* embryo

## Abstract

**Background:**

Canonical Wnt signals, transduced by stabilized β-catenin, play similar roles across animals in maintaining stem cell pluripotency, regulating cell differentiation, and instructing normal embryonic development. Dysregulated Wnt/β-catenin signaling causes diseases and birth defects, and a variety of regulatory processes control this pathway to ensure its proper function and integration with other signaling systems. We previously identified GTP-binding protein 2 (Gtpbp2) as a novel regulator of BMP signaling, however further exploration revealed that Gtpbp2 can also affect Wnt signaling, which is a novel finding reported here.

**Results:**

Knockdown of Gtpbp2 in *Xenopus* embryos causes severe axial defects and reduces expression of Spemann-Mangold organizer genes. Gtpbp2 knockdown blocks responses to ectopic Wnt8 ligand, such as organizer gene induction in ectodermal tissue explants and induction of secondary axes in whole embryos. However, organizer gene induction by ectopic Nodal2 is unaffected by Gtpbp2 knockdown. Epistasis tests, conducted by activating Wnt signal transduction at sequential points in the canonical pathway, demonstrate that Gtpbp2 is required downstream of Dishevelled and Gsk3β but upstream of β-catenin, which is similar to the previously reported effects of Axin1 overexpression in *Xenopus* embryos. Focusing on Axin in *Xenopus* embryos, we find that knockdown of Gtpbp2 elevates endogenous or exogenous Axin protein levels. Furthermore, Gtpbp2 fusion proteins co-localize with Dishevelled and co-immunoprecipitate with Axin and Gsk3b.

**Conclusions:**

We conclude that Gtpbp2 is required for canonical Wnt/β-catenin signaling in *Xenopus* embryos. Our data suggest a model in which Gtpbp2 suppresses the accumulation of Axin protein, a rate-limiting component of the β-catenin destruction complex, such that Axin protein levels negatively correlate with Gtpbp2 levels. This model is supported by the similarity of our Gtpbp2-Wnt epistasis results and previously reported effects of Axin overexpression, the physical interactions of Gtpbp2 with Axin, and the correlation between elevated Axin protein levels and lost Wnt responsiveness upon Gtpbp2 knockdown. A wide variety of cancer-causing Wnt pathway mutations require low Axin levels, so development of Gtpbp2 inhibitors may provide a new therapeutic strategy to elevate Axin and suppress aberrant β-catenin signaling in cancer and other Wnt-related diseases.

**Electronic supplementary material:**

The online version of this article (doi:10.1186/s12964-016-0138-x) contains supplementary material, which is available to authorized users.

## Background

Wnt signaling regulates various cell behaviors including proliferation, differentiation, polarization and migration, and is required to generate embryonic polarity as well as maintain normal tissue homeostasis [1–4]. Inappropriate activation of Wnt signaling in ventral tissues during early development can lead to the formation of a secondary axis in a variety of embryos [1, 5], and inappropriate Wnt target gene induction is thought to contribute to a broad range of neoplasms [4, 6, 7] including the majority of hereditary colorectal cancers [7]. Moreover, besides cancers, mutations in Wnt signaling components are common in many diseases and contribute to birth defects [8], yet therapeutics directed at the Wnt pathway remain limited [9].

Central to the mechanism of canonical Wnt signaling is the stabilization of a cytoplasmic pool of β-catenin protein, which results in its subsequent nuclear translocation and induction of target genes. In the absence of Wnt ligand, cytoplasmic β-catenin is phosphorylated by glycogen synthase kinase-3 β (Gsk3b) as part of the multisubunit destruction complex that also includes the Adenomatous Polyposis Coli (Apc) and Axin proteins. Phosphorylated β-catenin is recognized by the F-box protein, β-transducin repeat-containing protein (β-TrCP), part of an E3 ubiquitin ligase that catalyzes β-catenin ubiquitination and proteasomal degradation [10]. Upon Wnt ligand binding to Frizzled and Lrp5/6 receptors, focal accumulations of the Disheveled protein occur along with the phosphorylation of the Lrp5/6 tail by Gsk3b and Casein Kinase-1 (Ck1), which results in the subsequent recruitment and inhibition of the destruction complex, and concomitant stabilization of β-catenin [11–13].

Relative to other Wnt pathway components, Axin protein is maintained at low endogenous levels and imparts a rate-limiting step in β-catenin targeting by the destruction complex [14, 15]. Overexpression of Axin is sufficient to block β-catenin stabilization and target gene expression, and can block primary axis formation as well as ectopic secondary axis formation in *Xenopus* embryos triggered by overexpression of Wnt ligand or a kinase-dead form of Gsk3b (dnGsk3b) [1, 5].

Although the precise regulation of Axin turnover is not yet fully understood, several aspects of its degradation and regulation have recently been uncovered. In the presence of Wnt ligand, Axin protein is targeted for degradation while *axin2* expression is itself transactivated, allowing for initial promotion and subsequent negative feedback of signaling [16, 17]. More recently, Axin protein has been recognized to undergo PolyADP-ribosylation (PARsylation) via Tankyrases 1 and 2 (Tnks1/2), which promotes subsequent Axin ubiquitination by the Ring Finger 146 (Rnf146) E3 ubiqutin ligase, prompting proteasomal degradation of Axin [18–20].

In this study we have examined the requirements for GTP binding protein 2 (Gtpbp2), a largely uncharacterized protein that is distantly related to the Eef1a (Ef1α) family of large GTPases [21, 22], in the regulation of embryonic Wnt signaling. We find that morpholino knockdown of Gtpbp2 results in axial patterning defects and reduced induction of organizer genes in *Xenopus* embryos. Furthermore, we demonstrate that Gtpbp2 is required for transduction of Wnt signaling and negatively correlates with Axin protein levels. Our findings reveal a new member involved in the regulation of proteins that transduce canonical Wnt signals, and illuminate Gtpbp2 as a potential drug target for diseases involving disregulated Wnt signaling.

## Results

### Gtpbp2 knockdown disrupts axial patterning and reduces induction of β-catenin target genes

**Fig. 1:**
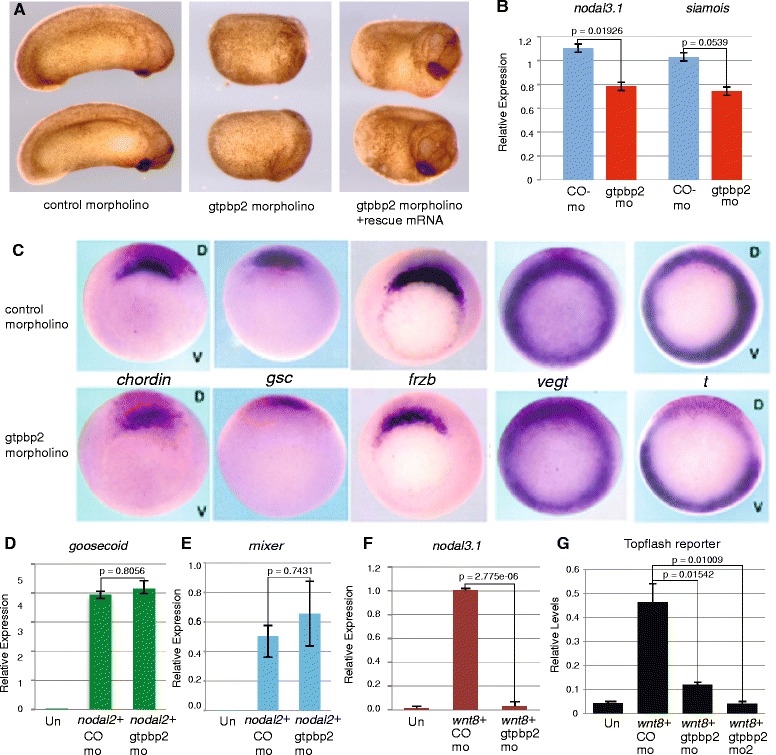
Gtpbp2 is required for Wnt but not nodal target gene induction. **a** Four-cell *Xenopus* embryos injected dorsally with 25 ng of Gtpbp2 MO (*middle*, 93 % abnormal, *n* = 94), but not a control 5-base mismatch MO (*left*, 95 % normal, *n* = 54), generated tadpoles with severe axial defects that were partially rescued by co-injection of the MO and a cocktail of 0.5 ng *gtpbp2a* and 50 pg *gtpbp2b* mRNA (*right panel*, 56 % rescued head and anterior structures, *n* = 78). **b** Embryos injected bilaterally at the 2-cell stage with 15 ng Gtpbp2 (gtpbp2 mo) or mismatch control (co-mo) morpholino were cultured until stage 10.25 then measured for *nodal3.1*, *siamois*, and *ornithine decarboxylase (odc)* levels. Amounts of *nodal3.1* or *siamois* were normalized to *odc* and relative levels shown as mean ± s.e.m of *n* = 3 with p-values from *t*-test. **c** Expression of organizer genes *chordin*, *gsc*, and *frzb* as well as the pan-mesodermal markers *t* was severely reduced in embryos injected with Gtpbp2 morpholino into two dorsal blastomeres at the four-cell stage. Expression of another T-box gene *vegt* was not affected. Experiments were repeated three times. **d**-**f** mRNAs encoding *nodal2* (10 pg; **d**, **e**) or *wnt8* (10 pg, **f**) were coinjected with control or Gtpbp2 morpholino into the animal pole of two cell embryos. Animal caps were cut at midblastula (stage 8), and the expression of nodal targets *goosecoid* (**d**) and *mixer* (**e**), and the Wnt8 target *nodal3.1* (**f**), were measured using qPCR at early gastrula stage 10.5. Relative expression levels shown as mean ± s.e.m (*n* = 3); p-values from *t*-test. **g** Knockdown of Gtpbp2 in caps treated with *wnt8* showed that Gtpbp2 is required for induction from a Wnt/β-catenin reporter. Embryos were injected with combinations of 10 pg *wnt8*, 4 ng GFP, 30 ng Gtpbp2 m1, Gtpbp2 m2 or control morpholinos as indicated, along with 100 pg of Super Topflash and 60 pg of TK-Renilla Luciferase plasmids. Reporter activities were normalized to *renilla* luciferase activity from a co-injected TK-RL construct and shown as mean ± s.e.m (*n* = 3) with p-values from *t*-test

We identified the large GTPase, Gtpbp2, as a novel Smad1 interactor as part of a yeast two-hybrid screen to identify new components of the BMP signaling pathway [23]. Our subsequent work demonstrated that Gtpbp2 is indeed required for normal regulation of Smad1/BMP target genes and ventral patterning in *Xenopus* embryos, and it is expressed in a tissue specific manner [24]. However, we observed particular developmental abnormalities resulting from Gtpbp2 inhibition in *Xenopus* embryos that could not be explained by defects in BMP signaling. The phenotypes of morpholino-injected embryos (morphants) included reduced head structures when translation-blocking morpholinos were targeted to dorsal blastomeres (Fig. 1a). Identically-injected morphant embryos showed reduced expression of Spemann-Mangold organizer genes *siamois*, *nodal-related 3 (nodal3.1*), *chordin, and goosecoid* (by qPCR; Fig. 1b, Additional file 1) and *chordin, goosecoid, and frzb1* (by whole mount in situ hybridization; Fig. 1c). The expression of the pan mesodermal T-box gene *t* (brachyury) was also severely reduced, while the expression of another mesodermal T-box gene, zygotic *vegt,* was not affected in those morphants (Fig. 1c).

### Gtpbp2 is required for canonical Wnt signaling

The *Xenopus* embryonic organizer is induced via the intersection of several signaling pathways, including Wnt/β-catenin and Nodal/Smad2/3 [3, 25]. Since Gtpbp2 can engage the BMP/Smad1 pathway, we hypothesized that Gtpbp2 might regulate nodal signals. However, knockdown of Gtpbp2 by injection of a translation-blocking morpholino (M1 of reference 24 unless noted otherwise) had no effect on Activin/Nodal direct target gene expression (*goosecoid*, *mixer*) in pluripotent *Xenopus* blastula ectodermal explants (animal caps) (Fig. 1d, e). The organizer genes effected in whole embryos by dorsal Gtpbp2 knockdown, above, can be directly (*nodal3.1* [26], *siamois* [27]) or indirectly (*goosecoid*, *chordin* [28, 29]) activated by Wnt/β-catenin. Therefore, we tested whether Gtpbp2 is required for induction of the Wnt direct-response genes, *nodal3.1* and *siamois* in animal caps. Embryos were treated with Wnt8 ligand (as injected mRNA) and either control or Gtpbp2-specific morpholinos, and animal caps were excised and scored for the induction of *nodal3.1* or *siamois*. Wnt8 failed to induce either gene upon Gtpbp2 knockdown (Fig. 1f, and Fig. 4a discussed below). Similarly, induction of the β-catenin-responsive TOPflash reporter by *wnt8* in animal caps was inhibited by knockdown of Gtpbp2, by two different morpholinos (Fig. 1g), and knockdown of Gtpbp2 reversed the stabilization of β-catenin in response to *wnt8* in animal caps (Additional file 2).

### Gtpbp2 binds to Axin and Gsk3b

**Fig. 2:**
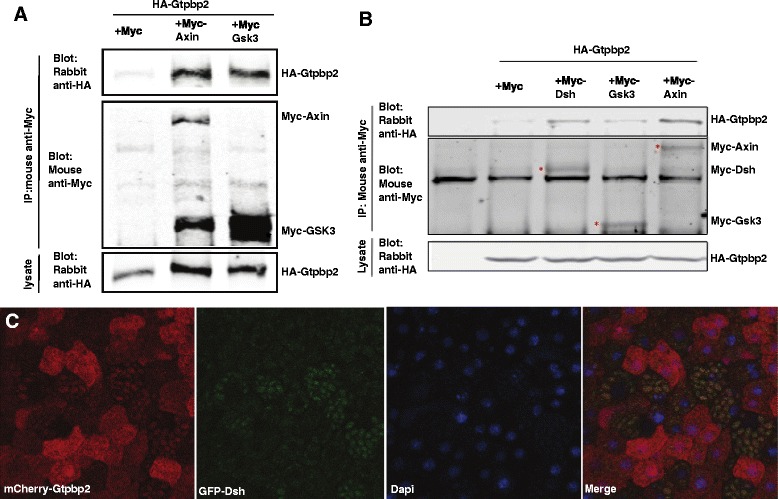
Gtpbp2 associates with components of the β-catenin destruction complex. **a** HA-Gtpbp2 was coexpressed in Hek293t cells with empty vector (Myc), Myc-Axin or Myc-Gsk3b and lysates were immunoprecipitated with anti-myc antibody followed western blot assay to detect precipitated Myc-tagged proteins and co-precipitated HA-Gtpbp2. The expression HA-Gtpbp2 in cells was confirmed by western blot on cell lysates. **b** mRNAs encoding HA-Gtpbp2 were injected into two-cell *Xenopus* embryos along with mRNAs encoding either the Myc tag, Myc-Axin, Myc-Gsk3b, or Myc-Gsk3b, as indicated. Embryos were lysed at gastrula stage 11, and were immunoprecipitated with anti-myc antibody followed western blot assay to detect precipitated Myc-tagged proteins and co-precipitated HA-Gtpbp2. HA-Gtpbp2 expression in cells was evaluated by western blot on cell lysates. **c** mCherry-Gtpbp2 relocalizes to GFP-Dvl2 containing granules when co-expressed in *Xenopus* animal caps (scored at late blastula, stage 9). mCherry-Gtpbp2 distribution is diffuse in cells lacking GFP-Dsh

To further investigate the potential role of Gtpbp2 in Wnt signaling, we examined whether Gtpbp2 could interact with Wnt pathway components. Our results show that tagged *Xenopus* Gtpbp2 protein co-immunoprecipitated with tagged components of the β-catenin destruction complex, Gsk3b and Axin, in human cells (Fig. 2a). Similar results were observed in proteins expressed in frog embryos, as tagged Axin and Disheveled proteins (and weakly Gsk3b) co-immunoprecipitated with tagged Gtpbp2 (Fig. 2b).

We also examined the subcellular localization of tagged forms of Gtpbp2 in the presence or absence of coinjected *dishevelled* mRNA by injecting one cell of a two cell embryo with mRNA encoding mCherry-Gtpbp2, and the other cell with mRNAs encoding mCherry-Gtpbp2 and GFP-Dishevelled. We previously reported a membrane localization for mCherry tagged Gtpbp2, however injecting a lower dose of mRNA (500 pg instead of 2 ng) resulted in a more general cytoplasmic localization (Fig. 2c), consistent with the staining pattern of a Gtpbp2 antibody on human cells described in the Human Protein atlas [26]. However, when co-expressed with GFP-tagged Disheveled, Gtpbp2 localized to cytoplasmic foci along with Dishevelled (Fig. 2c).

### Gtpbp2 is required for inactivation of the β-catenin destruction complex

**Fig. 3:**
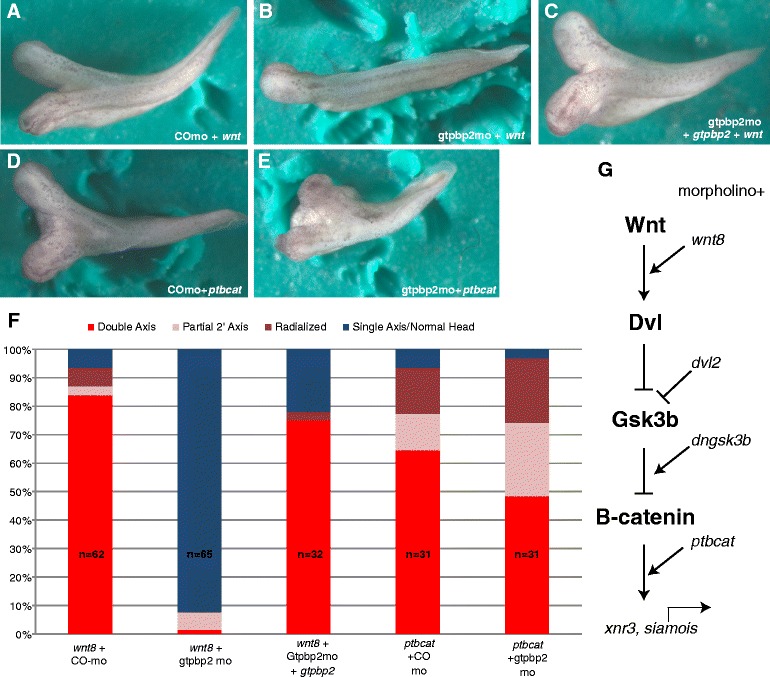
Gtpbp2 is required for ectopic axis induction by Wnt but not stabilized β-catenin. **a**-**e** Axis induction phenotypes. **a**, **c** Control or **b**, **d**-**e** Gtpbp2 morpholinos (25 ng) were injected into both ventral blastomeres at the 4 cell stage, and mRNAs encoding (**a**-**c**), Wnt8 (4 pg) or **d**-**e** ptBcat (25 pg) were injected into a single ventral vegetal blastomere at the 8–16 cell stage. The Gtpbp2 morpholino blocks induction of secondary axes from *Xenopus* Wnt8 but not by a phospho-resistant form of β − catenin (ptBcat). **e** Coinjection of Gtpbp2 morpholino along with morpholino-resistant *gtpbp2* mRNA (1 ng) restored secondary axes in Wnt-injected embryos. **f** Counts of total injected phenotypes, including intermediate Wnt phenotypes (partial secondary axis or enlarged head). **g** Diagram showing epistatic relationships between Wnt pathway reagents analyzed here and in Fig. 4

To complement animal cap assays, we used a phenotypic test to examine whether Gtpbp2 is required for the formation of secondary axes induced by overexpressing Wnt pathway components (Fig. 3). Induction of canonical Wnt signaling by injection of positive regulators of the Wnt signaling pathway (or dominant negative forms of negative regulators) can induce a secondary body axis in the ventral side of *Xenopus* embryos^,^ or activate Wnt-responsive target genes in animal cap assays [1, 5]. We found that Gtpbp2 knockdown blocked the formation of secondary axes induced by *wnt8* mRNA overexpression in a ventral blastomere resulting in a majority of normal tadpoles (Fig. 3a, b). The ability of Wnt8 to induce secondary axes was restored when the Gtpbp2 morpholino was co-injected with *gtpbp2* mRNA resistant to the Gtpbp2 morpholino (Fig. 3c). However, we found that the Gtpbp2 morpholino had no effect on the formation of secondary axes by a stabilized form of β-catenin (*ptbcat)* that lacks Gsk3b target residues and thus avoids targeting by the destruction complex (Fig. 3e). These data (quantified in Fig. 3f) suggest that Gtpbp2 is required for Wnt signaling at the level of the destruction complex.

**Fig. 4:**
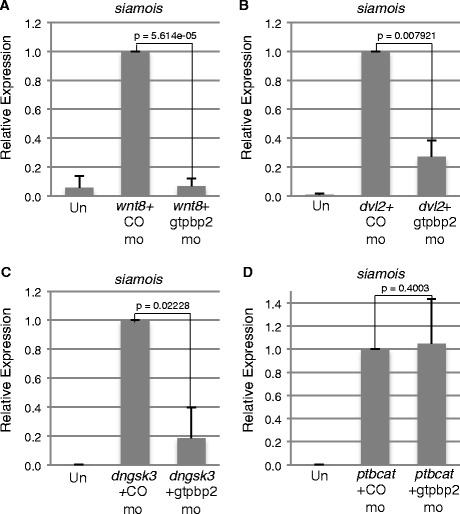
Gtpbp2 epistasis experiments suggest Gtpbp2 works at the level of Axin turnover. **a**-**d** mRNAs encoding activators of the Wnt pathway, including **a** *wnt8*, **b** *disheveled (dvl2)*, **c** *kinase-dead Gsk3b (dnGsk3)* or **d** *phospho-resistant ß-catenin* (*ptbcat*) respectively were coinjected with 40 ng control or Gtpbp2 morpholino into the animal pole of two cell *Xenopus* embryos. Animal caps were excised at blastula stage 8, and the levels of *siamois* transcript were measured using qPCR at early gastrula stage 10.5 and shown as mean ± s.e.m of *n* = 3. The Gtpbp2 morpholino blocks induction from *wnt8*, *disheveled,* or dominant negative *dngsk*3 (**a**-**c**), but not from a phospho-resistant β-catenin (*ptbcat*) (**d**)

To further examine how Gtpbp2 works to positively regulate Wnt signaling, we tested epistatic relationships between Gtpbp2 and Wnt signal transduction pathway components, using isolated *Xenopus* blastula animal caps. Using reagents that engage the Wnt pathway at different levels (Fig. 3g), we found that Gtpbp2 knockdown in caps (Fig. 4) reduced the induction of *siamois* by ectopic wnt8, disheveled *(dvl2)*, and dominant-negative kinase-dead Gsk3b (*dnGsk3b*) (Fig. 4 a-c). However, Gtpbp2 knockdown had no effect on *siamois* induction by *ptbcat* (Fig. 4d)*,* consistent with our results from the secondary body axis assay.

### Gtpbp2 knockdown leads to increased Axin protein levels

**Fig. 5:**
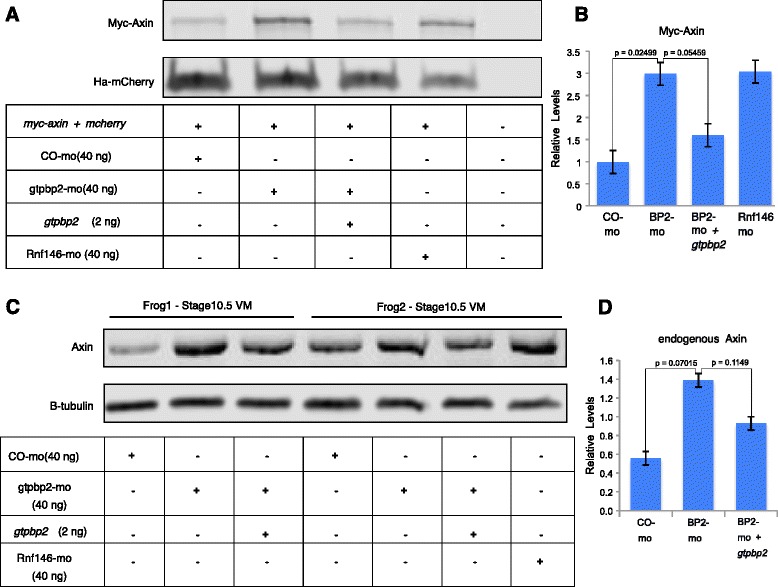
Gtpbp2 reduces Axin protein levels in *Xenopus* embryos. **a** *HA-mcherry* and *myc-axin* mRNAs were co-injected with a morpholino (40 ng) or morpholino + *gtpbp2b* mRNA (2 ng), into the marginal zone of two cell embryos, and proteins were analyzed by western blot. **b** Quantitation of myc-Axin levels, shown as mean ± s.e.m of *n* = 3. **c** Morpholino (40 ng) or morpholino + *gtpbp2* mRNA (2 ng) plus rhodamine-dextran tracer were injected in the marginal zone of two ventral blastomeres of 4 cell stage embryos. The rhodamine-dextran was used to facilitate ventral marginal zone (primarily mesoderm) dissections under a florescent dissection scope at early gastrula stage 10.5, and two of three biological replicate western blots are shown. **d** Quantitation of endogenous Axin shown as mean ± s.e.m of *n* = 3

**Fig. 6:**
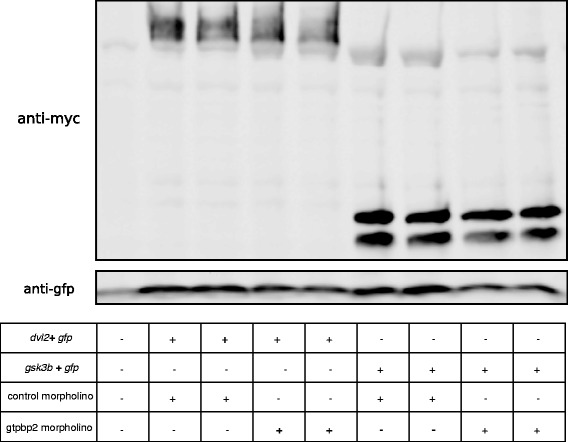
Loss of Gtpbp2 does not affect stability of Dishevelled or Gsk3b. *Xenopus* embryos were co-injected in the marginal zone of two-cell blastomeres with 1 ng of mRNA encoding *myc-Dvl2* or *myc-Gsk3b* fusion proteins and 250 ng of *gfp* mRNA (as loading control), along with 40 ng of control or Gtpbp2 morpholino. Embryos were lysed at stage 10.5, run on an 8 % PAGE gel, and western blots were with probed with monoclonal mouse anti-myc 9E10 antisera. Duplicate lanes correspond to independent biological replicates

Our epistasis and interaction experiments point to an inhibitory role for Gtpbp2 on the activity of the β-catenin destruction complex. Axin is a rate-limiting component in Wnt-signaling, being maintained at low steady-state protein levels yet required for efficient targeting of β-catenin by Gsk3b [14, 15]. Accumulation of Axin protein can overcome the competitive inhibition of endogenous Gsk3b by ectopic kinase dead Gsk3b [5]. Therefore, we hypothesized that Gtpbp2 might be affecting Wnt signaling at the level of the destruction complex via negative regulation of Axin protein levels. We examined the effect of Gtpbp2 loss of function on Axin protein levels by co-injecting embryos with Gtpbp2 morpholinos and an mRNA encoding myc-tagged Axin. As a positive control, we designed a morpholino against the Ring Finger 146 ubiquitin ligase (Rnf146), knockdown of which has been demonstrated to stabilize Axin levels in cultured mammalian cells [19, 20]. We found that levels of myc-tagged Axin increased almost three-fold when embryos were injected with either of two morpholinos targeting Gtpbp2 (M1 or M2) [24], or the MO targeting Rnf146, compared to the control MO (Fig. 5a, b; Additional file 3). Axin stabilization was reversed when Gtpbp2 morpholino M1 was co-injected with morpholino-resistant *gtpbp2* mRNA (Fig. 5). No change was seen in the levels of tagged Gsk3b or Dvl2 when co-injected with Gtpbp2 morpholino compared to control (Fig. 6), pointing to a specific effect on Axin.

To test whether Gtpbp2 could affect endogenous Axin levels in the embryo, we used an antibody raised against *Xenopus* Axin [17] to measure changes in Axin protein expression in early gastrula (stage 10.5) ventral mesoderm explants. We targeted this tissue because it has endogenously high levels of Wnt activity at this point in development [27]. Similar to the effects we observed on ectopic myc-Axin, knockdown of both Gtpbp2 and Rnf146 caused increases in endogenous Axin levels in ventral mesoderm explants (Fig. 5c, d).

## Discussion

These studies are the first report of a role for Gtpbp2 in Wnt signaling and provide initial insights into the biochemical roles of this rather enigmatic GTPase. Our data show that Gtpbp2 is required for transduction of canonical Wnt signaling in *Xenopus* embryos and embryo explants. Inhibiting Gtpbp2 represses dorsoanterior axis formation and expression of Spemann-Mangold organizer genes, and while head loss is dramatic, the effects on organizer markers is somewhat mild, which may be due to maternal Gtpbp2 protein not effected by the morpholino. Indeed, our previous report showed a high level of maternal *gtpbp2* transcripts in the egg [24], so cleavage stage knockdown of Gtpbp2 is likely to be partial. However, our present results clearly demonstrate that gtpbp2 morphant embryos, and zygotic wnt target genes in animal cap explants, show a near complete lack of response to ectopic induction of Wnt signaling.

**Fig. 7:**
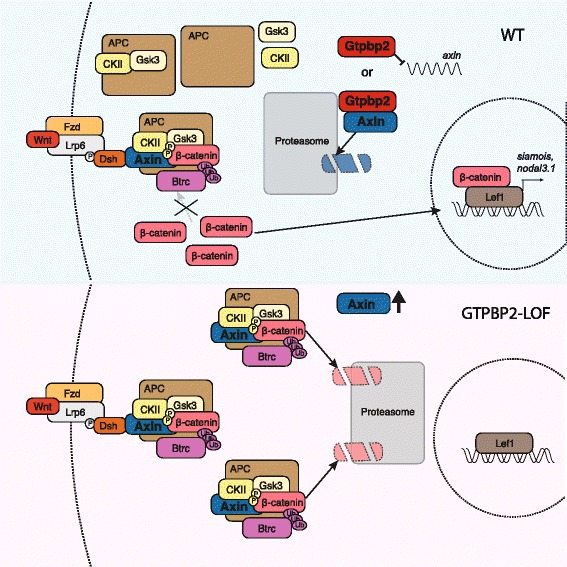
Gtpbp2 is required for Wnt signaling via regulation of Axin levels. In the presence of Gtpbp2 (*top*), Axin protein is maintained at low levels, and all active destruction complexes can be inactivated in the presence of Wnt ligand, allowing for accumulation of cytoplasmic β-catenin. However, when Gtpbp2 levels are reduced (Loss of Function; *bottom*), Axin levels rise and can form additional destruction complexes with ample free Gsk3b, CkII, and APC, either allowing for additional complexes to saturate the pool of cytoplasmic β-catenin and/or allowing some complexes to escape Wnt regulation and continue to degrade β-catenin. Gtpbp2 may regulate Axin protein by direct engagement of free Axin, destruction complex-bound Axin (with proteasomal targeting), or potentially other mechanisms (e.g., mRNA translation)

Our results support a model (Fig. 7) that Gtpbp2 functions in Wnt signaling by maintaining low levels of Axin protein. Quantitative models of Wnt signal transduction point to Axin as the most concentration-limited component of the destruction complex, with Axin protein being approximately 2500 times less abundant than Gsk3b and 5000 times less abundant then either Apc or Dishevelled in *Xenopus* egg extracts [14, 15]. Axin also is the rate-limiting factor in the degradation of β-catenin, as addition of nanomolar amounts of Axin protein can cause a 5–10 fold increase in the degradation rate of β-catenin [14]. Since we found that Gtpbp2 could regulate the levels of Axin provided ectopically by mRNA injection, we can infer that Gtpbp2 regulates Axin levels post-transcriptionally. A mouse mutation in *gtpbp2* causes brain degeneration when combined with a tRNA mutation [30], indicating Gtpbp2 can function in general translation regulation, likely by clearing paused ribosomes. Although we cannot rule out a role for Gtpbp2 in regulating translation from, or stability of, *axin* mRNA, such regulation would have to occur through elements in the coding sequence or 3′ UTR, as injected myc-axin lacks the endogenous 5′ UTR. Since we observe specific effects on the levels of Axin only, but not other endogenous or ectopically expressed proteins, Gtpbp2 in this *Xenopus* embryonic context seems unlikely to be regulating general mRNA translation. Furthermore, the interaction of Gtpbp2 with Axin and other destruction complex components suggests the regulation of Axin by Gtpbp2 occurs at the level of Axin turnover.

Several factors have been identified which promote or suppress Axin turnover. Axin protein degradation is promoted by active Wnt signaling in response to the phosphorylation of the cytoplasmic tail of Wnt co-receptor Lrp6 [17, 31]. However, subsequent studies have demonstrated that Lrp6-mediated degradation of Axin is not required to initiate canonical Wnt signaling [12, 13], but this could be a means of promoting chronic Wnt stimulation. More recently it has been recognized that Axin protein undergoes Poly-ADP-ribosylation (PARsylation) via Tankyrases 1 and 2 (Tnks1/2) [18], which promotes Axin ubiquitination by the Rnf146 E3 ubiqutin ligase and consequent proteasomal degradation [19, 20]. Additionally, Axin also can be destabilized by ubiquitination by the Smurf2 ubiquitin ligase [32], or stabilized by Gsk3b phosphorylation [33], sumoylation [34], or by the ubiquitin-specific protease Usp34 [35].

Detailed investigation into how Gtpbp2 affects post-translational modifications, such as sumoylation, ubiquitination, phosphorylation, and/or PARsylation, of Axin or other proteins will likely provide more insight into the specific molecular mechanism of the Gtpbp2 protein, and resolve the apparent differences in Gtpbp2 function in *Xenopus* and mouse embryos.

Furthermore, because Gtpbp2 is required for normal Wnt signaling and operates downstream of proteins which engage the destruction complex, such as Apc and Lrp5 [5, 6] that are commonly mutated in Wnt-driven cancers, we suggest Gtpbp2 as a potential drug target for cancers and other diseases involving excessive Wnt signaling.

Recent work has identified crosstalk between the Wnt and BMP signaling pathways [36–39]. Smad1, a key component of the BMP signal transduction pathway, is also a target of Gsk3b. Like β-catenin, Smad1 is stabilized in response to active Wnt signaling and Gsk3b inhibition. As Gtpbp2 knockdown shows reductions in both Wnt and BMP signaling, one intriguing possibility for a common mechanism would be that the increased levels of Axin protein in Gtpbp2 morphants may also affect Smad1 levels. Although Wnt and Gsk3b regulate Smad1 levels, Axin’s role in this process is unclear. Axin has been shown to bind and regulate Smad3, however the authors of that paper mention unshown data failing to identify similar interactions between Axin and Smad1 [40]. Therefore future studies that tease out the role of Axin and Gtpbp2 may illuminate the convergent and divergent features of Gtpbp2 in these key signaling pathways.

## Conclusions

In summary, we have found that Gtpbp2 is a novel regulator of Wnt signaling in *Xenopus* embryos. We find that morpholino knockdown of Gtpbp2 results in axial patterning defects and reduced induction of organizer genes in *Xenopus* embryos. Furthermore, we demonstrate that Gtpbp2 is involved in the negative regulation of Axin protein levels, itself a negative regulator of Wnt signaling. Our findings reveal new insights into the tight proteolytic regulation of proteins involved in transducing canonical Wnt pathway signals, and illuminate Gtpbp2 as a potential drug target for diseases involving dysregulated Wnt signaling.

## Methods

### General reagents

Gtpbp2 and control morpholinos were as previously described [24], and a morpholino targeting the translation start site of both *Xenopus rnf146* homeologs was designed for this study, 5′-GCTAACCTCCCCACAACCAGCCATC -3′(GeneTools). Plasmids for *gtpbp2* were previously described [24], and remaining plasmids were generous gifts from Ken-ichi Takemaru (Stony Brook University), Peter Klein (University of Pennsylvania) and Sergei Sokol (Mount Sinai School of Medicine), including XE10 pCS2-Xwnt-8 (*wnt8)* [40], pCS2-xnr2 (*nodal2)* [41], pCS2MT-xDsh (*myc-dsh)* [42]*,* Xg134 pCS2MT-Gsk3 (*myc-gsk3*) [43]*,* Xg137 pCS2-dnGsk3 *(dngsk3)* [44]*,* XE49 pCS2MT-ptβ − catenin (*ptbcat)* [45], XE43 pCS2-GFP-Xdsh (*gfp-dvl2)* [46]*,* pCS2-Axin MtFu1 [5] (*myc-axin*). Synthetic mRNA was generated in vitro from linearized plasmids using AmpliCap™ SP6 High Yield Message Maker Kit (Cellscript). Reporter assays were conducted using the thymidine kinase promoter-Renilla luciferase (pRL-TK, Promega) and M50 Super 8x TOPFlash plasmids [46]. Antibodies were grown from monoclonal supernatants mouse anti-Myc (9E10, 1:100), a gift from P. Klein (rabbit anti-*Xenopus* Axin 1066, 1:1000) [17], or purchased from the following: rabbit anti-HA (1:500) (RHGT-45A), ICL; mouse anti-β-tubulin (BYA-6068, 1:20,000), Accurate Chemical and Scientific Corporation; anti-GFP (B-2, 1:200), Sigma; iRDye800 conjugated affinity purified anti-mouse IgG (goat) (610-131-121, 1:5000), Rockland Immunochemicals; Alexa-fluor 680 goat anti-rabbit (A-21076, 1:5000), Molecular Probes.

### Embryo microinjection and dissection

*Xenopus laevis* husbandry, microinjection and animal cap dissections were done as described previously [14]. For the ventral mesoderm explants, Alexa-488 Dextran 10,000 (Molecular Probes, 5 mg/ml) was included in the injected solutions to facilitate ventral marginal zone (primarily mesoderm) dissections under a Zeiss Discovery V8 florescent dissection scope. *Xenopus laevis* were obtained as a non-specific wild type strain from *Xenopus* Express and Nasco Inc., and maintained in cycled colony.

### Quantitative PCR and in situ hybridization

Embryos or explants were treated as indicated and cDNA was generated as previously described [14]. Real-time quantitative PCR was conducted on a Roche lightcycler 480 with SYBR Green I Master (Roche), using primers sequences and conditions as described [47, 48, 49]. Target gene expression was normalized to levels of *odc* transcripts, and statistical analyses were conducted using R. Whole mount in situ RNA hybridization (WISH) was performed as described [14].

### Immunoprecipitation and western blotting

Hek293t cells were co-transfected using linear PEI (1 mg/ml) with plasmids encoding HA-Gtpbp2, Myc, Myc-Gsk3b, and/or Myc-Axin. Cells were incubated overnight and lysed in NP40 lysis buffer (10 mM Tris-Cl pH 8.0, 137 mM NaCl, 10 % Glycerol, 1 % NP-40, + complete protease and phosphatase inhibitor tablets; Roche). Cell lysates were incubated with mouse anti-Myc (9E10) antibody overnight, purified using anti-IgG magnetic beads (NEB) according to the manufacturers instructions. Immunoprecipitations and whole cell lysates (1:10 of IP volumes) were blotted with mouse anti-myc (1:100) and rabbit anti-HA antibodies (1:500). For western blotting *Xenopus* embryos or explants were lysed in NP40 lysis buffer and extracted with an equal volume of 1,1,2-trichlorotrifluoro-ethane (Sigma), and then separated using SDS-page. Detection of western blots was conducted with IR secondary antibodies (Alexa 680 and IRdye800) scanned on a Licor Odyssey Classic Imager. Levels of ectopic myc-Axin were normalized to levels of co-injected HA-mCherry.

### Fluorescent microscopy

Two-cell stage *Xenopus* embryos were injected into the animal pole with *mcherry-gtpbp2* mRNA (500 pg) into both cells, with one cell co-injected with *gfp-dvl2* (500 pg), followed by imaging at late blastula (stage 9). Embryos were first examined live, then gently fixed in 1 % paraformaldehyde in PBS for 15 min, washed 3x in PBS, and costained with 2 mg/ml 4,6-diamino-2-phenylindole (DAPI). Embryos were then imaged on using a 10x objective on a Zeiss fluorescence microscope (Motorized Axio Imager Z1) with an ApoTome analysis unit.

### Reporter assays

Reporter assays were conducted by injecting *Xenopus* embryos with 100 pg Super TopFlash together with 50 pg pRL-TK reporter plasmids, and 10 pg of *xwnt8* mRNA, and 30 ng of gtpbp2 M1, M2, or control morpholino as indicated. Animal caps were cut at mid-blastula stage 8 and cultured until mid-gastrula stage 11, at which point cell extracts were prepared and analyzed using Dual-Glo Luciferase Assay Reporter Assays (Promega).

## Abbreviations

CK1, Casein-kinase 1; Dsh, Dishevelled; frzb, frizzled-b; gsc, goosecoid; Gsk3b, glycogen synthase kinase-3 β; Gtpbp2, GTP-binding protein 2; β-TrCP, beta-transducin repeat-containing protein; odc, ornithine decarboxylase

## Additional files

Additional file 1: GTPBP2 regulates the expression of additional organizer markers. (PDF 45 kb) Additional file 2: Gtpbp2 morphants impair β-catenin stabilization in response to wnt8. (PDF 222 kb) Additional file 3: Stabilization of Axin occurs with two separate Gtpbp2 morpholinos. (PDF 416 kb)
