# Supplementary material for: Gtpbp2 is a positive regulator of Wnt signaling and maintains low levels of the Wnt negative regulator Axin
Source: Cell Commun Signal. 2016 Aug 2;14:15. doi: 10.1186/s12964-016-0138-x (PMC4969687; doi:10.1186/s12964-016-0138-x)
Supplement: Additional file 1: — GTPBP2 regulates the expression of additional organizer markers. (PDF 45 kb) [file 12964_2016_138_MOESM1_ESM.pdf]

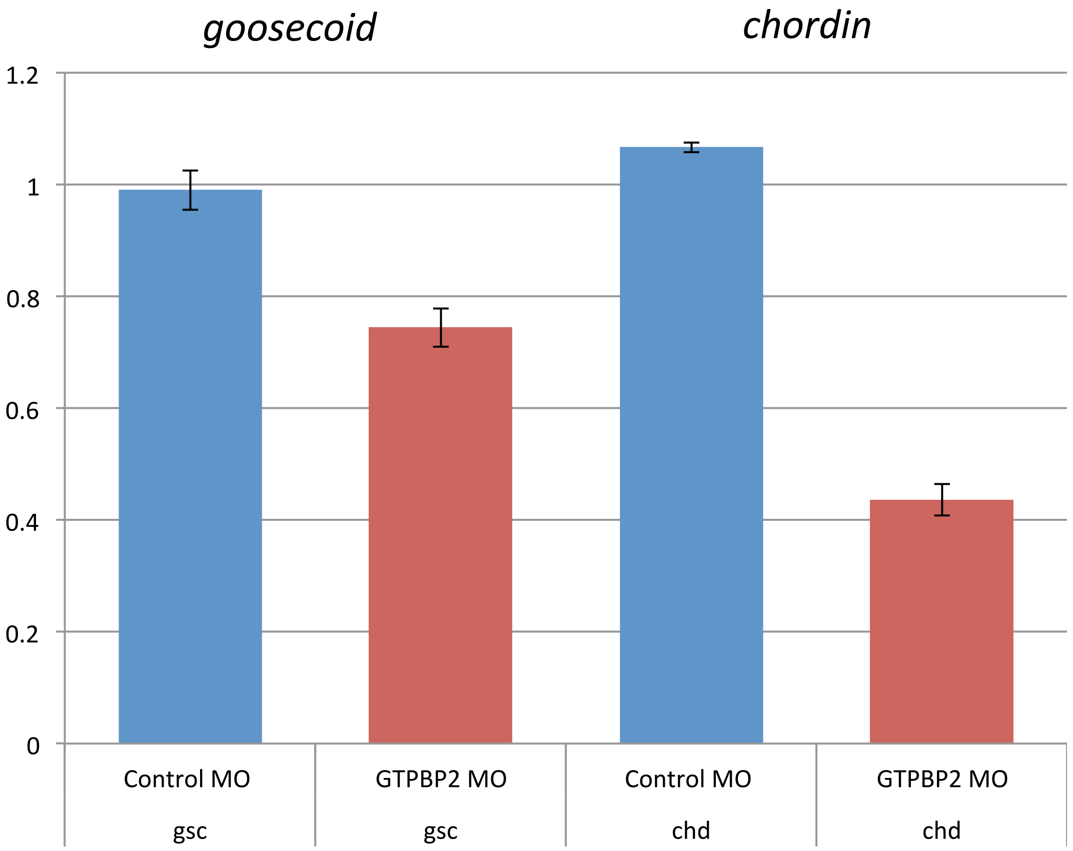

Additional File 1: GTPBP2 regulates the expression of additional organizer markers. Embryos injected bilaterally at the 2-cell stage with 15 ng Gtpbp2 (gtpbp2 mo) or mismatch control (co-mo) morpholino were cultured until stage 10.25 then measured for *nodal3.1*, *siamois*, and *odc* levels. Amounts of *goosecoid (gsc)* or *chordin (chd)* were normalized to *odc* and relative levels shown as mean ± s.e.m of n = 3.
