# Supplementary material for: Gtpbp2 is a positive regulator of Wnt signaling and maintains low levels of the Wnt negative regulator Axin
Source: Cell Commun Signal. 2016 Aug 2;14:15. doi: 10.1186/s12964-016-0138-x (PMC4969687; doi:10.1186/s12964-016-0138-x)
Supplement: Additional file 2: — Gtpbp2 morphants impair β-catenin stabilization in response to wnt8. (PDF 222 kb) [file 12964_2016_138_MOESM2_ESM.pdf]

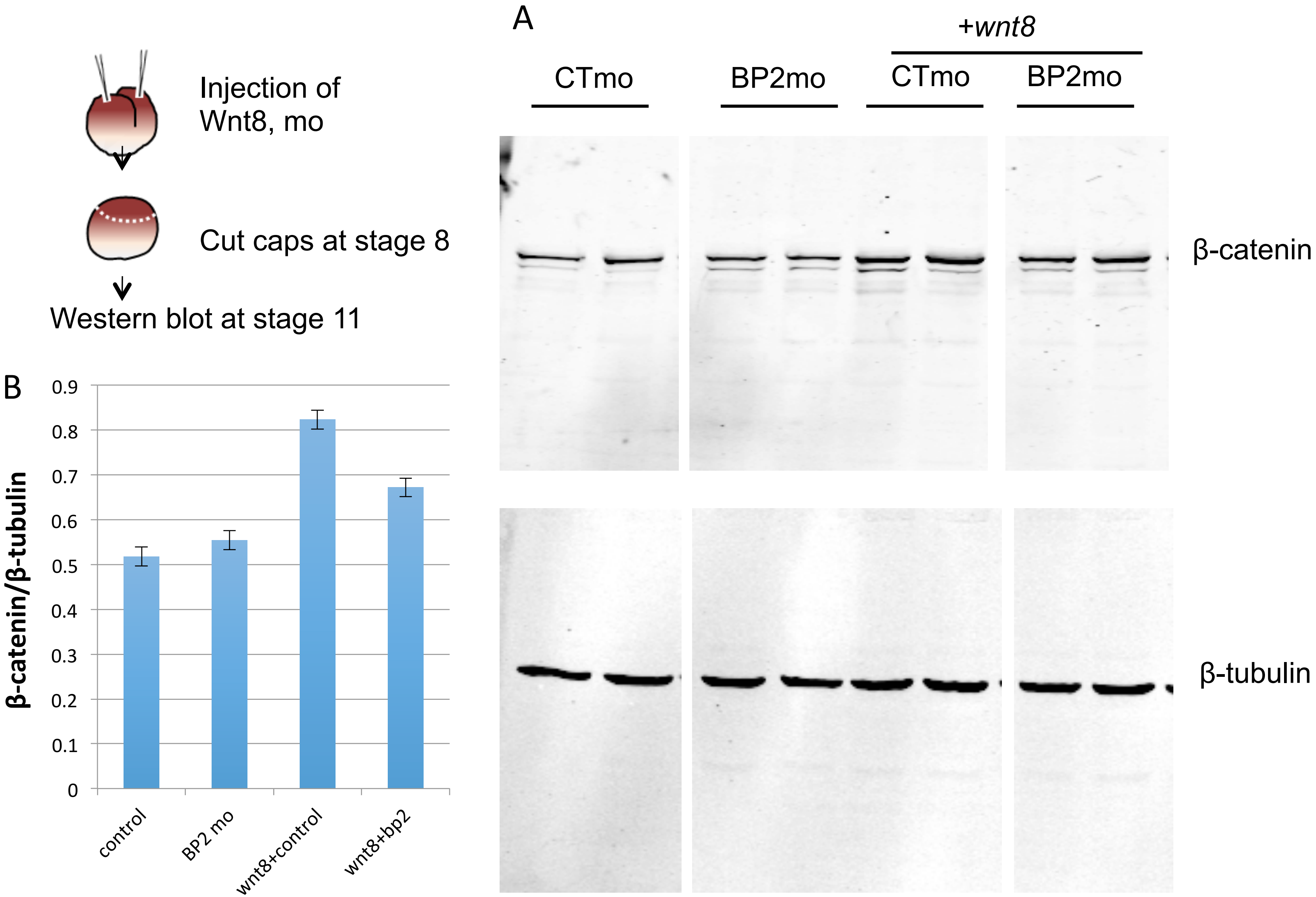


**Additional File 2*: Gtpbp2 morphants impair β-catenin stabilization in response to wnt8:*** *A)* The animal pole of 2-cell *Xenopus* embryos were injected with Gtpbp2 morpholino (BP2 mo), Control morpholino (CTmo), and *wnt8* as indicated. Animals caps were cut at stage 8 and cultured until stage 11, and analyzed via western blot for β-catenin and β-tubulin levels. B) Quantitation of relative β-catenin levels shown as mean ± s.e.m of n =2.
