# Supplementary material for: Gtpbp2 is a positive regulator of Wnt signaling and maintains low levels of the Wnt negative regulator Axin
Source: Cell Commun Signal. 2016 Aug 2;14:15. doi: 10.1186/s12964-016-0138-x (PMC4969687; doi:10.1186/s12964-016-0138-x)
Supplement: Additional file 3: — Stabilization of Axin occurs with two separate Gtpbp2 morpholinos. (PDF 416 kb) [file 12964_2016_138_MOESM3_ESM.pdf]

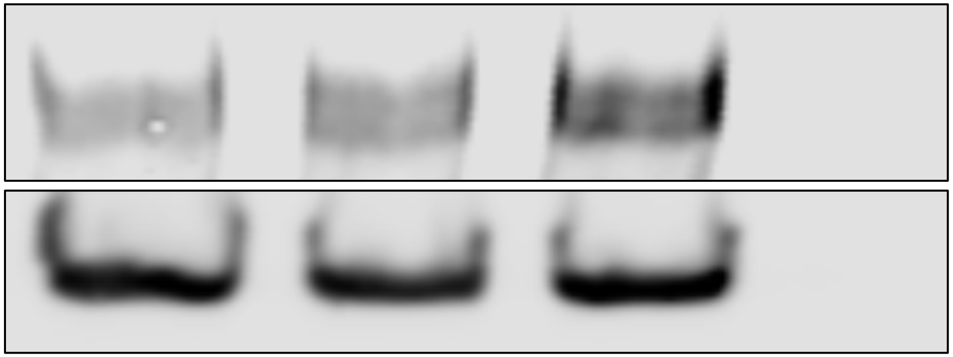

HA-mCherry+ Myc-Axin	+	+	+	-
5xMM control morpholino	+	-	-	-
GTPBP2 M1 morpholino	-	+	-	-
GTPBP2 M2 morpholino	-	-	+	-

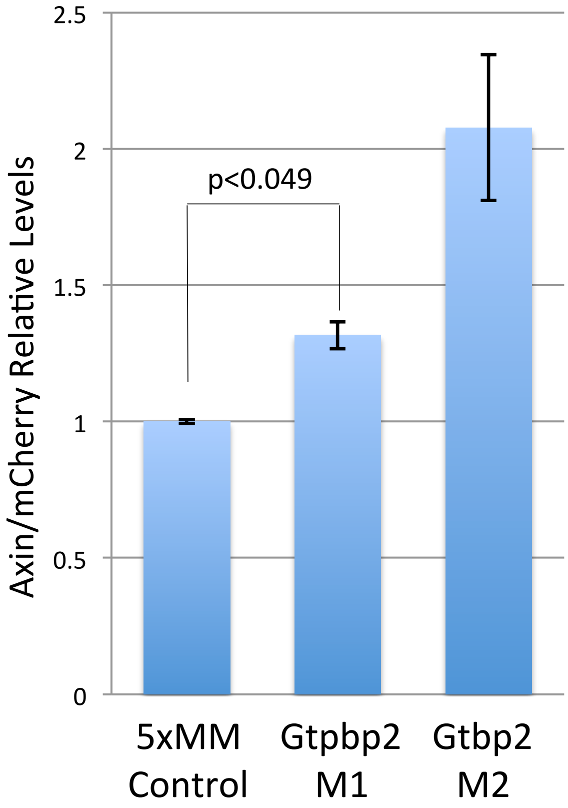

Additional File 3: Stabilization of Axin occurs with two separate Gtpbp2 morpholinos
A) HA-mcherry and *myc-axin* mRNAs were co-injected with mRNAs encoding HA-mCherry (1ng) and myc-Axin (1 ng) and either 5x mismatch (5xMM) control, Gtpbp2 M1, or Gtpbp2 M2 morpholinos (40 ng) at the two cell stage, and tagged proteins were detected via western blot of stage 10.5 protein lystates. B) Quantitation of relative myc-Axin levels shown as mean ± s.e.m of n =4.
